# The Effect of Electroacupuncture on Osteosarcoma Tumor Growth and Metastasis: Analysis of Different Treatment Regimens

**DOI:** 10.1155/2013/387169

**Published:** 2013-10-21

**Authors:** Branden A. Smeester, Mona Al-Gizawiy, Elaine E. O'Brien, Marna E. Ericson, Jennifer L. Triemstra, Alvin J. Beitz

**Affiliations:** ^1^Department of Medicinal Chemistry, College of Pharmacy, University of Minnesota, Minneapolis, MN 55455, USA; ^2^Department of Radiology, Medical College of Wisconsin, Milwaukee, WI 53226, USA; ^3^Department of Veterinary and Biomedical Sciences, College of Veterinary Medicine, University of Minnesota, St. Paul, MN 55108, USA; ^4^Department of Dermatology and Center for Drug Design, University of Minnesota, Minneapolis, MN 55455, USA; ^5^Department of Veterinary and Biomedical Sciences, University of Minnesota, Room 205 Veterinary Medicine Building, 1971 Commonwealth Avenue, St. Paul, MN 55108, USA

## Abstract

Osteosarcoma is the most common malignant bone tumor found in children and adolescents and is associated with many complications including cancer pain and metastasis. While cancer patients often seek complementary and alternative medicine (CAM) approaches to treat cancer pain and fatigue or the side effects of chemotherapy and treatment, there is little known about the effect of acupuncture treatment on tumor growth and metastasis. Here we evaluate the effects of six different electroacupuncture (EA) regimens on osteosarcoma tumor growth and metastasis in both male and female mice. The most significant positive effects were observed when EA was applied to the ST-36 acupoint twice weekly (EA-2X/3) beginning at postimplantation day 3 (PID 3). Twice weekly treatment produced robust reductions in tumor growth. Conversely, when EA was applied twice weekly (EA-2X/7), starting at PID 7, there was a significant increase in tumor growth. We further demonstrate that EA-2X/3 treatment elicits significant reductions in tumor lymphatics, vasculature, and innervation. Lastly, EA-2X/3 treatment produced a marked reduction in pulmonary metastasis, thus providing evidence for EA's potential antimetastatic capabilities. Collectively, EA-2X/3 treatment was found to reduce both bone tumor growth and lung metastasis, which may be mediated in part through reductions in tumor-associated vasculature, lymphatics, and innervation.

## 1. Introduction

There has been a dramatic increase in the use of complementary and alternative medicine approaches to treat cancer patients suffering from oncological pain, fatigue and the side effects of chemotherapy [1–5], despite limited research available to support their use [6]. While there are a number of studies and reviews that support [2] or question [7] the use of acupuncture for treating cancer symptoms, few studies have examined the use of acupuncture to treat the cancer itself [8]. Moreover, there are no studies to date that have investigated sex differences in acupuncture's effect on cancer. Since acupuncture has been shown to have both immunoregulatory and anti-inflammatory effects, acupuncture could potentially affect tumor growth by inducing changes in the immune system and/or by producing direct effects on tumor associated inflammation [9–12]. In this regard, we have recently reported that EA can reduce tumor-associated inflammation [13].

Osteosarcoma is a highly malignant cancer generally occurring in early adolescence [14], and while improvement in survival rates has increased over the last 30 years, osteosarcoma patients face poor prognoses. Adjuvant chemotherapy and surgical resection are common therapies [14, 15], but treatment efficacy is poor. Osteosarcoma metastasis is common with nearly 90% of patients having metastatic lesions, particularly in the lungs [16, 17]. Although our understanding of the mechanisms underlying tumor angiogenesis [18, 19], tumor progression [20–23], and metastasis [14, 17] is improving, the complex nature of the bone tumor microenvironment presents unique challenges in identifying novel drug targets. While many studies have examined drug effects on these mechanisms, the potential for alternative medicine approaches, particularly acupuncture, to affect cancer growth and metastasis has not been explored.

 Emerging research indicates that acupuncture can alter angiogenesis [24], raising the possibility that acupuncture may alter tumor vasculature, which ultimately could affect tumor progression and metastasis. The present study was designed to examine the potential effect of different electroacupuncture (EA) treatment schedule regimens on osteosarcoma tumor growth and pulmonary metastases and to determine whether sex differences exist in EA's effects. Here we hypothesized that low-frequency EA has antitumor growth and antimetastatic effects, by inducing alterations in tumor-associated vasculature, lymphatics, and innervation densities. To test this hypothesis, we utilized a hind paw osteosarcoma mouse model developed at the University of Minnesota [25] to examine the above relationships *in vivo*.

## 2. Methods and Materials

### 2.1. Cells

The K7M2 cell line was a kind gift from Dr. Khanna et al. [26] and was derived from the K7 cell lines initially established from a spontaneous murine osteogenic sarcoma [27]. Cells were grown and maintained in accordance with standard cell culturing techniques. K7M2 cells were grown to 80–90% confluence in 75 cm^2^ flasks (Corning, Lowell, MA) in Dulbecco's Modification of Eagles Medium (Invitrogen, Carlsbad, CA) containing 1% penicillin/streptomycin and 10% Fetal Bovine Serum prior to implantation. Cell cultures were housed in a water-jacketed incubator with 5% carbon dioxide at 37°C.

### 2.2. Animals

The present study used a well-established mouse hind paw model of bone cancer [25, 28] to examine the effects of EA on tumor growth and metastasis. In this model osteosarcoma cells are implanted into the calcaneous bone of the heel of a mouse as outlined below. A total of 67 female and 100 male young adult BALB/c mice (National Cancer Institute, Bethesda, MD) were used in this study as summarized in Table 1. The inbred BALB/c mouse strain is syngeneic to the osteosarcoma cells used. Animals were maintained on a 12 hr light/dark cycle with food and water *ad libitum*. Procedures were performed in accordance with the guidelines recommended by the International Association for the Study of Pain, and all experimental protocols were approved by the Animal Care and Use Committee at the University of Minnesota.

### 2.3. Implantation

Cells were prepared for implantation by first pouring off the culture medium and washing with phosphate-buffered saline (PBS). Trypsin was added to the flask and placed in an incubator for 3 min. Upon detachment, cells were suspended in an ample amount of culture medium to terminate enzymatic activity and centrifuged for 10 min at 1,000 g. Osteosarcoma cells were resuspended in a known amount of PBS for counting, quantified on a hemacytometer, and pelleted and resuspended in PBS. Osteosarcoma cells were resuspended to a final concentration of 1 × 10^6^ cells in 50 *μ*L. Initially, animals were anesthetized in a plexiglass chamber using 3% isoflurane in 3 L/min oxygen. Prior to the injection procedure, we assessed anesthetic depth by examining the loss of purposeful voluntary movement, loss of a blink reflex, muscle relaxation, and loss of response to reflex stimulation (toe or tail pinch with firm pressure). Once each mouse was completely anesthetized, a maintenance rate of 2% isoflurane in 1.5 L/min oxygen was maintained during the short implantation procedure. Tumor cells were manually injected by boring into the calcaneus bone using a 29 gauge needle connected to a sterile 0.3 mL insulin syringe as previously described [25]. A control group of BALB/c mice (*n* = 10) received an injection of saline into the calcaneus bone rather than tumor cells. These mice were used for the initial behavioral testing. Following injection, mice were allowed to recover in cages on a heating pad. Animals showing any signs of dysfunction (e.g. problems with ambulation, lethargy, or excessive bleeding) or any animals in which the tumor subsequently did not grow were euthanized and removed from the study. This occurred in less than 5% of the animals used in this study.

### 2.4. Tumor Growth

Tumor growth was measured using calipers at PID 0, 3, 7, 10, 14, 17, and 21. Baseline measurements were established prior to tumor implantations. The investigator performing the tumor growth measurements was blinded to the treatment (Tumor-EA versus Tumor-Sham EA versus No Tumor-No EA, i.e., saline implantation).

### 2.5. Electroacupuncture Method and Treatment Regimens

 For EA or sham-acupoint treatment, animals were initially anesthetized with 3% isoflurane/3 L oxygen and then maintained at 2% isoflurane/1.5 L oxygen for the length of the EA or sham procedure (30 min). For the EA and sham-EA acupuncture groups, two stainless steel intradermal needles (SEIRIN America, Weymouth, MA) were inserted to a depth of 3 mm into the hind limb at the ST-36 Zusanli acupoint located between the tibia and fibula, approximately 5 mm lateral to the anterior tubercle of the tibia. Rather than testing multiple acupoints and examining their effects on tumor nociception, we focused on one point, ST-36, for the purposes of this study. This allowed us to evaluate differences in the frequency and timing of the EA application, rather than the effect of stimulating different individual acupoints or simultaneous stimulation of multiple acupoints. The ST-36 Zusanli acupuncture point was selected because it has been used to evaluate the effect of EA in rodent models of cancer pain [13] and inflammatory pain [29] and in treating cancer [8]. In the EA groups, the ST-36 Zusanli acupoint was stimulated using a Maxtens 1000 dual channel stimulator with a 4 Hz pulse rate, 100 *μ*s pulse width, for a total of 30 min as previously reported [13]. 

 To our knowledge, there are no controlled studies in the literature that have examined the effects of different acupuncture regimens (treatment parameters) on tumor growth and metastasis. The present study examines and compares the potential antitumor/anti-metastasis effects of six different EA treatment regimens administered at the ST-36 acupoint in a rodent hind paw model of bone cancer. These six regimens were used in a previous study from our laboratory to evaluate their effect on cancer pain [13]. The six treatment protocols were as follows: EA administered once weekly starting at PID 7 (EA-1X/7); EA administered twice weekly starting at PID 3 (EA-2X/3), PID 5 (EA-2X/5), PID 7 (EA-2X/7); EA administered once on day 1 after tumor cell implantation (EA Once/1); and EA administered 3 times prior to tumor cell implantation with no treatment after implantation, which we designated as the prophylactic (EA Pro) treatment subgroup. Osteosarcoma tumor-bearing animals were divided into the following three experimental and control groups: *Group 1*: an EA treated tumor group, which consisted of 6 subgroups of animals all implanted with tumor cells but each receiving a different EA treatment regime as described above (EA-1X/7, EA-2X/3, EA-2X/5, EA-2X/7, EA Once/1, or EA Pro); *Group 2*: a sham-EA treated tumor group, which consisted of 6 subgroups of animals all implanted with tumor cells but each receiving a different sham-EA treatment regime (similar to the EA treatment regimens described above) that involved implanting an acupuncture needle into ST-36 but not applying any electrical current (Sham-1X, Sham-2X/3, Sham-2X/5, Sham-2X/7, Sham Once/1, and Sham Pro); *Group 3*: a Tumor-No EA treatment group (mice that were implanted with tumor cells but received no EA or sham treatment and day 21were subsequently euthanized at postimplantation; *n* = 10).

### 2.6. Transcardiac Perfusions

To prepare mouse tissues for immunohistochemical (IHC) studies, mice were deeply anesthetized with 50 mg/kg sodium pentobarbital (Nembutal; Ovation Pharmaceuticals, Inc., Deerfield, IL) injected intraperitoneally. When mice were no longer responsive to paw pinch, the thoracic cavity was quickly accessed via the abdomen to isolate the heart. A 21-gauge butterfly catheter (Terumo Medical Corporation, Somerset, NJ) was inserted into the left ventricle and secured using forceps. The right atrium was punctured to allow both drainage of blood and fixative. Fifteen mL of ice cold PBS was perfused followed by 30 mL of Zamboni's Fixative at a rate of 3 mL/min. Following perfusion, tumors were excised, postfixed in the same fixative overnight at 4°C and cryoprotected in 30% sucrose for 24–48 hours at 4°C prior to tissue sectioning. 

### 2.7. Immunohistochemistry on Tumor Sections

Tumors were then sectioned and immunostained with antibodies that specifically recognize blood vasculature, nerves, or lymphatics according to previously described protocols from our laboratories [25, 30]. Briefly, following fixation in Zamboni's fixative for 24 hr, tissues were transferred to 30% sucrose cryoprotection buffer. Tissues were mounted in Optimal Cutting Temperature (OCT, Electron Microscopy Sciences) and cut on a cryostat into 150 *μ*m thick sections. Sections were kept in PBS and washed in TX/PBS (0.3% Triton-X100 in PBS) prior to beginning the immunostaining procedure. The tissue was first incubated in 5% normal serum in TPBS (5% NTX/PBS, Jackson Immuno, West Grove, PA) overnight at room temperature under continuous slow rotation conditions using a gyrorotatory shaker. The tumor sections were then incubated overnight at room temperature in the following antibodies: (a) rat anti-CD31 (Biogenesis, Inc., Kingston, NH), which stains blood vessels; (b) rabbit anti-PGP9.5 (Biogenesis, Inc., Kingston, NH), which stains nerve fibers; or (c) goat anti-LYVE1 (Biogenesis, Inc., Kingston, NH), which stains lymphatics, diluted in 1% NTPBS. Sections were then washed 3X with 1% NTPBS over an 8-hour period. Following this washing regimen, the tissue sections were then incubated in secondary antibodies raised in donkey as follows: (a) anti-rat IgG conjugated to cyanine 2 (Cy2) and (b) anti-rabbit conjugated to cyanine 3 (Cy3) or anti-goat IgG conjugated to cyanine 5.18 (Cy5) (Jackson ImmunoResearch, West Grove, PA). The tumor sections were washed, and then individual sections were initially mounted in agar, dehydrated in ethanol, cleared with methyl salicylate, and then mounted using the colorless, neutral, xylene miscible mountant, DEPEX (Electron Microscopy Science, Poole, UK).

### 2.8. Histochemistry of Lung Sections

Following transcardiac perfusion, whole lungs were removed, postfixed in 10% neutral-buffered formalin (NBF) overnight at 4°C, and placed into 70% ethanol for paraffin embedding. Following a routine embedding procedure, 4 *μ*m thick paraffin sections were cut on a microtome, and the sections were subsequently stained with hematoxylin/eosin and visualized using light microscopy.

### 2.9. Quantification of Lung Metastasis Tissue

In order to compare the amount of metastases between tumor mice that received EA treatment twice per week and tumor mice that received no treatment, we examined the lungs of both groups at 40 and 60 days after mplantation both grossly and microscopically. For histological analysis, we quantified the area within the lungs occupied by tumor metastases using a modification of the methodological approach described by Väyrynen and coworkers [31]. Briefly, a total of 8 transverse lung tissue sections (4 *μ*m thick; spaced 300 *μ*m apart) through both lungs per animal were selected and scanned for the presence of metastases using light microscopy (Nikon, Melville, NY). Nikon ACT-1 (Nikon, Melville, NY) software was used to acquire all lung images for processing. To determine tumor quantity, all lung images were stacked and montaged together forming a complete photographic representation of the lung section, and then the number of metastases per section was quantified. Next, the area of the lungs occupied by metastatic tumor was measured per section of lung and totaled per animal using MetaMorph software (Molecular Devices, Sunnyvale, CA).

### 2.10. Quantification of PGP9.5, CD31, and LYVE1 Immunoreactivity

To quantify PGP9.5-, LYVE1-, and CD31-immunoreactivity in both the tumor core and in the subcutaneous tissue at the tumor periphery, we used a morphometric analysis approach as described in detail previously [30]. The tumor core consisted of the central core of the tumor within the calcaneus bone and histologically was composed primarily of a high density of malignant K7 M2 cells that displayed anisocytosis and were poorly organized architecturally and produced osteoid. It should be noted that as the tumor progresses the tumor cells invade and break down the bone and begin to migrate into the surrounding soft tissue. In the present analysis, the tumor periphery consisted of fascia outside the main tumor area that contained connective tissue, muscle, and some tumor cells. Single-photon laser scanning confocal microscopy (LSCM) was used to image mouse hind paw sections in the green channel (CD31-ir; blood vessels), red channel (PGP9.5-ir; nerve fibers), and far-red channel (LYVE1-ir; lymphatics) on the same tissue section. LSCM serial optical section data sets were then collected using identical parameters: 150 *μ*m thick *z* stacks were collected at 200x magnification of the mouse hind paw. Using Adobe Photoshop CS3 and ImageJ software, total area pixel count of the entire selected periphery and tumor core was obtained. The average area pixel count of the CD31-ir, PGP9.5-ir, and LYVE1-ir was calculated by first thresholding the respective gray-scale images to reduce the contribution of nonspecific background signal. The immunopositive pixels were again selected, and a threshold pixel count was acquired for each channel. For each mouse, four nonoverlapping fields of view (periphery and core) for each antibody were obtained and analyzed.

### 2.11. Statistical Analysis

Complete statistical analyses of all data sets were carried out. Comparisons between groups were performed using either a one-way or two-way ANOVA with post hoc comparisons using Bonferroni's method. For single time point comparisons between groups or within a group, an unpaired Student's *t*-test was employed. Data was graphed using Prism 5.0 (GraphPad Software, La Jolla, CA). The level of significance was set at *P* ≤ 0.05.

## 3. Results

### 3.1. Calcaneal Implantation Causes Robust Tumor Development

**Figure 1:**
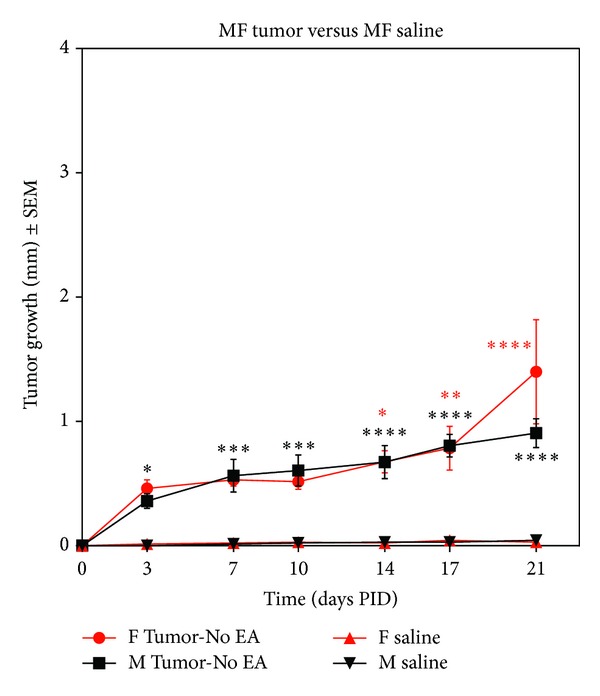
Graph illustrating tumor growth from postimplantation day (PID) 3 to 21 in male and female mice as measured using calipers. Pronounced tumor growth is evident as early as PID 3 and extends through PID 21 in both male and female animals (*n* = 9–11/group) as compared to their saline (no tumor) controls (*n* = 7/group, *P < 0.05, **P < 0.01, ***P < 0.001, and ****P < 0.0001).

Implantation of osteosarcoma cells into the calcaneus of BALB/c mice produced robust tumor growth that is evident as early as PID 3 in males and PID 14 in females (Figure 1; *P < 0.05, **P < 0.01, ***P < 0.001, ****P < 0.0001) as compared to their saline injected controls. While there appears to be tumor growth in female animals at days 7 and 10, this growth did not reach statistical significance until day 14. Tumor growth was sustained in both male and female mice throughout the duration of the experiment.

### 3.2. Effect of Different Treatment Regimens: EA Treatment at ST-36 Significantly Alters Tumor Growth

#### 3.2.1. EA-1X/7 (EA Administered Once per Week Beginning at PID 7)

**Figure 2:**
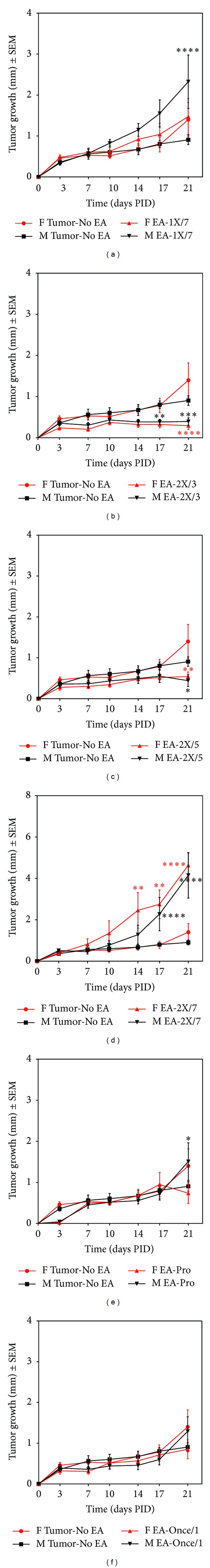
Graphs illustrating the effects of electroacupuncture on tumor growth ((a)–(f)) using the following six treatment schedules: (a) EA given once per week beginning on PID 7 (EA-1X/7); (b) EA given twice per week beginning on PID 3 (EA-2X/3); (c) EA given twice per week beginning on PID 5 (EA-2X/5); or (d) PID 7 (EA-2X/7); (e) three EA treatments given prior to implantation (EA-Pro); and (f) EA given once on PID 1 (EA-Once/1). EA-1X/7 treatment resulted in increased tumor growth that was more robust in males (M) versus their female (F) and control counterparts ((a); *n* = 8–11/group). EA-2X/3 produced the longest lasting antitumor growth effects of all the EA regimens tested for both male and female animals ((b); *n* = 8–11/group). EA-2X/5 provided late antitumor effects beginning at PID 21 ((c); *n* = 7–11/group). Conversely, EA-2X/7 elicited a marked increase in tumor growth, which began earlier in female animals ((d); *n* = 7–11/group). EA-Pro also elicited a protumor growth response at PID 21 that was only significantly different in males. ((e), *n* = 8–11/group). Neither EA-Once/1 or any of the sham treatment regimens had any effect on tumor growth and were comparable to their Tumor-No EA controls ((f), *n* = 7–11/group, sham not shown). Data shown as mean % response ± SEM, *P < 0.05, **P < 0.01, ***P < 0.001, and ****P < 0.0001 as compared to the Tumor-No EA control groups).

EA-1X/7 administered to the ST-36 acupoint produced a progressive increase in tumor growth rate in male tumor mice as compared to male tumor mice that did not receive EA treatment. This robust growth was most evident at PID 21 (Figure 2(a); ****P < 0.0001). Conversely, EA treatment given once a week had no effect on tumor growth in female mice at any time points tested (Figure 2(a); *P* < 0.05). Moreover, sham EA treatment given once per week beginning at PID 7 had no effect on tumor growth in either male or female mice (data not shown). 

#### 3.2.2. EA-2X/3 (EA Administered Twice per Week Beginning at PID 3)

In male mice, EA administered twice per week beginning at PID 3 resulted in a significant reduction in tumor growth that was first evident on PID 17 and continued through PID 21 as compared to the Tumor-No EA control group. Female mice exhibited a similar reduction in tumor growth with the most robust response beginning on PID 21 (Figure 2(b); **P < 0.01, ***P < 0.001, and ****P < 0.0001). By comparison, there was a significant reduction in male tumor growth that began at PID 17, while female mice exhibited a more significant decrease in tumor growth than males at PID 21. EA-2X/3 was the only treatment regime to produce significantly robust and lasting decreases in tumor growth beginning at PID 17. Sham-EA treatment given twice per week beginning at PID 3 had no effect on tumor growth (data not shown). 

#### 3.2.3. EA-2X/5 (EA Administered Twice per Week Beginning PID 5)

Similar to the effect of EA-2X/3, administering EA twice a week beginning PID 5 significantly reduced tumor growth in both males and females at PID 21 (Figure 2(c); *P < 0.05, **P < 0.01) when compared to their Tumor-No EA control groups. Sham EA-2X/5 had no effect on tumor growth (data not shown).

#### 3.2.4. EA-2X/7 (EA Administered Twice per Week Beginning PID 7)

In contrast to the results of EA-2X/3 and EA-2X/5 administration, EA treatment twice weekly starting at PID 7 significantly increased tumor growth in both male and female animals. This robust increase in tumor growth was evident at PID 14, 17, and 21 in females and PID 17 and 21 in males as compared to their Tumor-No EA control groups, respectively (Figure 2(d); **P < 0.01 and ****P < 0.0001). By contrast, Sham EA-2X/7 had no effect on tumor growth (data not shown). 

#### 3.2.5. EA-PRO (EA Administered 3 Times prior to Tumor Cell Implantation)

Early prophylactic treatment elicited a significant increase in tumor growth at PID 21, but only in male animals, as compared to their Tumor-No EA control group (Figure 2(e); *P < 0.05). EA-PRO had no effect on tumor growth in female animals at any time point tested (Figure 2(e); *P* > 0.05). Sham EA given prophylactically also had no effect on tumor growth (data not shown). 

#### 3.2.6. EA-ONCE/1 (EA Administered Once at PID 1)

EA treatment given once at PID 1 had no effect on tumor growth at any time points tested (Figure 2(f); *P* > 0.05). Similarly sham EA-ONCE/1 had no effects on tumor growth at any time points (data not shown).

#### 3.2.7. EA Controls

Administration of sham EA (Sham EA-1X/7, Sham EA-2X/3, Sham EA-2X/5, Sham EA-2X/7, Sham EA PRO and Sham EA-ONCE/1) had no significant effect on tumor growth at any time point tested (data not shown; *P* > 0.05). 

### 3.3. EA-Induced Changes in Hind Paw Lymphatics, Vasculature, and Innervation

Since EA-2X/3 elicited the most robust and longest-lasting decrease in tumor growth, we focused our analysis of the effect of EA on tumor lymphatics, vasculature and innervation in tumor animals treated with EA-2X/3 that were euthanized on PID 21. We quantified the densities of lymphatics, blood vessels and nerves in both the subcutaneous periphery surrounding the hind paw tumor as well as in the tumor core in EA-2X/3 and non-EA treated mice as described in the methods section. Moreover, since this antitumor growth effect was evident by PID 17 in males, but not until PID 21 in females, this set of experiments was only performed in male mice. In addition, because EA-2X/7 elicited the most robust and longest-lasting increase in tumor growth, we repeated these procedures in male EA-2X/7 and Tumor-No EA control animals as well.

**Figure 3:**
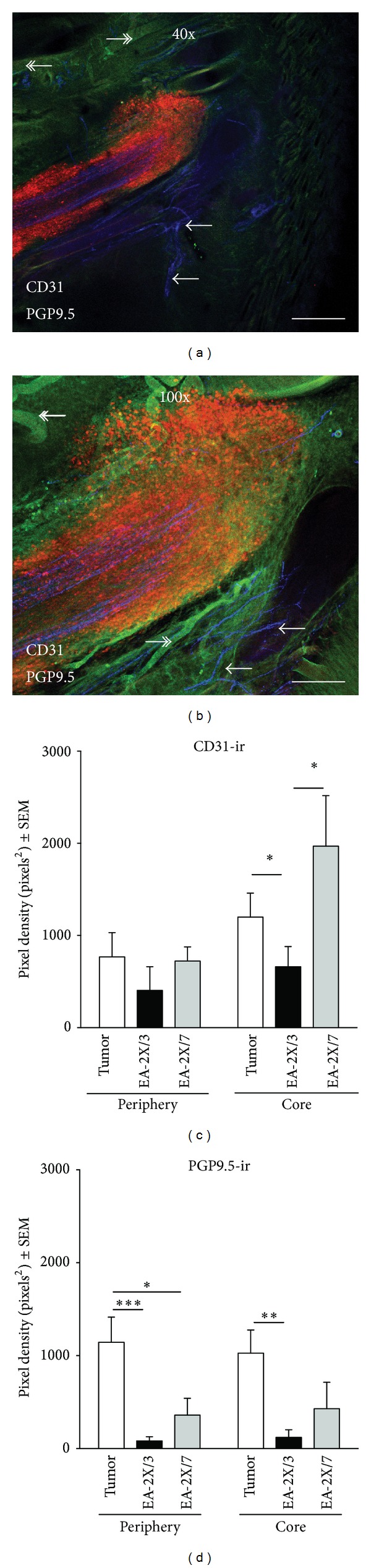
Representative photomicrographs of fluorescent immunohistochemical staining for blood vessels (CD31, green staining) and nerves (PGP9.5, blue staining) in the core (a) and periphery (b) of the hind paw tumor. Images were taken at 40x and 100x with an MRC 1024 Confocal/Multiphoton Imaging System (Bio-Rad, Hercules, CA). The scale bar in (a) represents 50 *μ*m, while the scale bar in (b) represents 20 *μ*m. Graphs illustrating the effect of electroacupuncture (EA) given twice per week beginning PID 3 (EA-2X/3) or PID 7 (EA-2X/7) on hind paw (c) vasculature and (d) innervation in male animals. EA-2X treatment produced a significant decrease in both blood vessel density and nerve fiber density in the core of the hind paw tumor as compared to the EA-2X/7 treated group and the Tumor-No EA control group (*n* = 6/group). Both EA-2X/3 and EA-2X/7 elicited significant reductions in innervation density in the periphery of the tumor, although EA-2X was more robust (*n* = 6/group). Data are shown as mean % response ± SEM, *P < 0.05, **P < 0.01, and ***P < 0.001 as compared to the Tumor-No EA control group. Positive CD31 (blood vessel) immunostaining is indicated by the double arrows (↞) in (a) and (b), while PGP9.5 (nerve fiber) immunostaining is indicated by single arrows (⟵).

**Figure 4:**
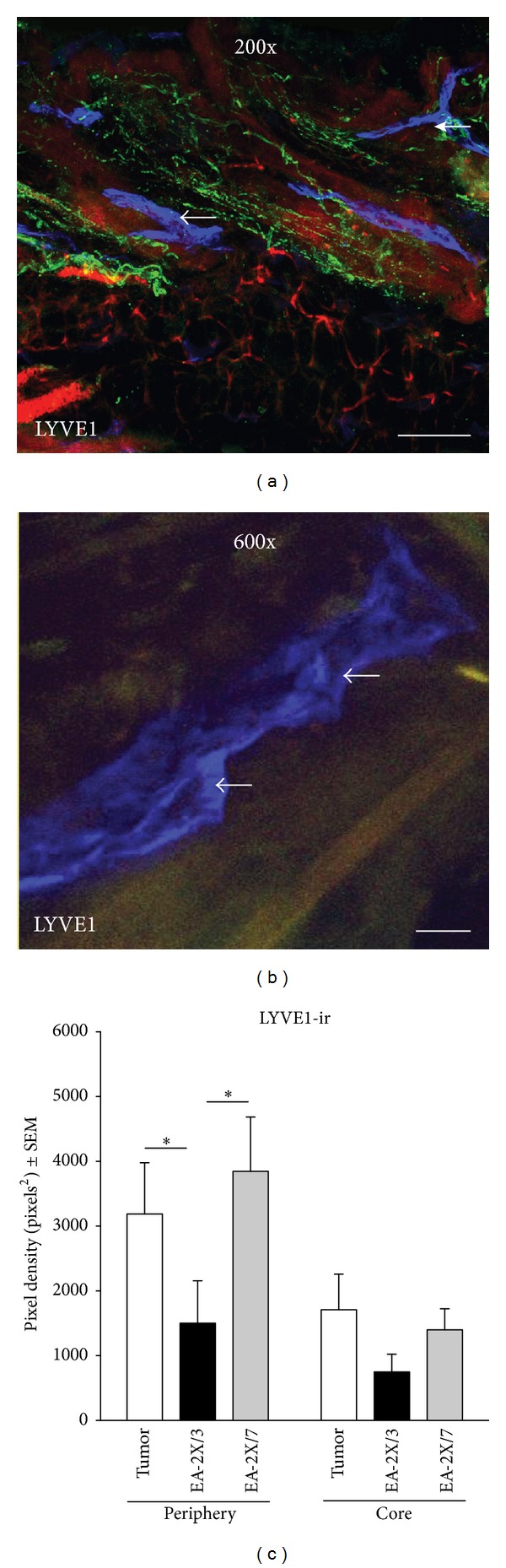
Representative photomicrographs of fluorescent immunohistochemical staining of lymphatics (LYVE1) in the hind paw tumor taken at 200x (a) and 600x (b), respectively. The images were captured with an MRC 1024 Confocal/Multiphoton Imaging System (Bio-Rad, Hercules, CA). Scale bars represent 50 *μ*m. (c) A graph showing the effect of EA-2X/3 and EA-2X/7 treatment on lymphatic immunostaining density at the tumor site. EA-2X/3, but not EA-2X/7, displayed a robust decrease in peripheral lymphatic density when compared to both its Tumor-No EA (TUM) and EA-2X/7 counterparts (*n* = 6/group). Positive LYVE1 (lymphatic) immunostaining is indicated by the single arrows.

In order to investigate potential mechanisms that might underlie acupuncture's effect on tumor growth, we utilized immunohistochemistry to quantify the densities of nerves, lymphatic vessels, and blood vasculature present in the subcutaneous periphery and tumor core of sections through the hind paw of either EA-2X/3 or EA-2X/7 treated animals and their Tumor-No EA control counterparts. No nonspecific staining was observed in any of the immunohistochemical control slides. Although quantitative differences in vasculature, lymphatics, and nerve fibers among EA-2X/3, EA-2X/7, and Tumor-No EA treated animals were detected with quantitative image analysis, such differences are not obvious in single photomicrographs, and thus, we have only included representative photomicrographs of the tumor site from Tumor-No EA treated animals in Figures 3 and 4 to illustrate the typical appearance of vessel, lymphatic, and nerve fiber immunostaining. 

#### 3.3.1. Blood Vessels (CD31-Ir)

EA-2X/3 treatment produced a significant decrease in CD31 immunostaining density in tumor core sections when compared to sections from either the Tumor-No EA or EA-2X/7 groups (Figures 3(a) and 3(c)—Core; *P < 0.05). Conversely EA-2X/3 or EA-2X/7 treatment had no significant effect on blood vessel density in the tumor periphery (Figures 3(b) and 3(d)—Periphery; *P* > 0.05). Image analysis measurements for average CD-31 density on PID 21 for Tumor-No EA, EA-2X/3, and EA-2X/7 in the periphery were 883 ± 289.5, 404 ± 257.1, and 723 ± 153.0, respectively; in the core the average CD-31 densities were 1404 ± 195.3, 661 ± 219.6, and 1969 ± 547.7, respectively.

#### 3.3.2. Nerve Fibers (PGP9.5-Ir)

Administration of either EA-2X/3 or EA-2X/7 treatment produced significant reductions in peripheral subcutaneous innervation (Figures 3(a) and 3(d)—Core; *P < 0.05, ***P < 0.001). Although EA-2X/7 treatment did not have a significant effect in nerve fiber density in the tumor core sections analyzed, EA-2X/3 significantly reduced the innervation density in the tumor core (Figures 3(b) and 3(d)—Periphery; **P < 0.01 for EA-2X/3, *P* > 0.05 for EA-2X/7). Image analysis measurements for average PGP9.5 density on PID 21 for Tumor-No EA, EA-2X/3, and EA-2X/7 in the periphery were 1326 ± 245.4, 83 ± 44.4, and 359 ± 182.0, respectively; in the core were 1158 ± 258.7, 121 ± 82.5, and 428 ± 286.2, respectively. 

#### 3.3.3. Lymphatics (LYVE1-Ir)

EA-2X/3 treatment produced a significant reduction in lymphatic density in the tumor periphery when compared to either the Tumor-No EA or EA-2X/7 groups (Figures 4(a) and 4(c)—Core; *P < 0.05). Image analysis measurements for average LYVE1 density on PID 21 for Tumor-No EA, EA-2X/3, and EA-2X/7 in the periphery were 3781 ± 639.1, 1501 ± 656.0, and 3846 ± 839.8, respectively, while in the tumor core the average LYVE1 densities for these same groups were 2014 ± 559.4, 749.0 ± 274.0, and 1400 ± 324.1, respectively. Though it did not reach statistical significance, there was a trend towards a reduction in lymphatics density in the tumor core of EA-2X/3 treated animals as well (Figures 4(b) and 4(c)—Core; *P* > 0.05).

### 3.4. Effect of EA-2X/3 on Bone Metastasis

**Figure 5:**
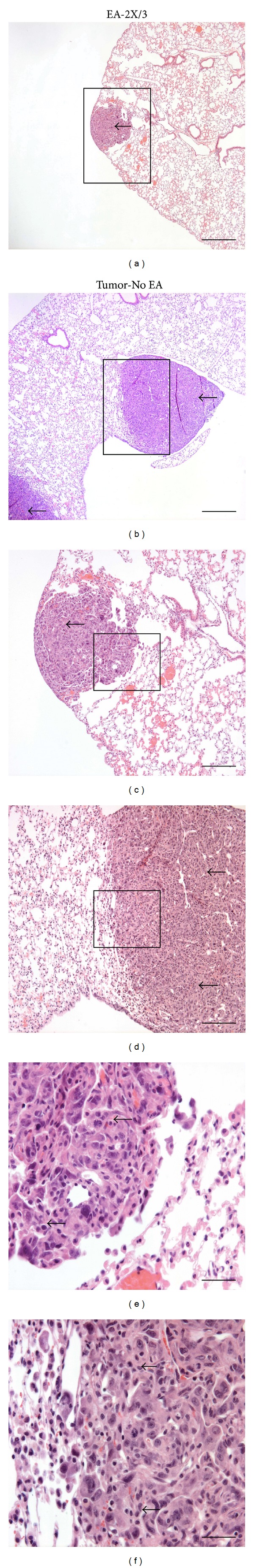
Representative photomicrographs of H&E stained lung sections showing the presence of visible pulmonary tumors (arrows) at ((a)-(b)) 4x, ((c)-(d)) 10x, and ((e)-(f)) 40x magnifications in animals receiving twice-weekly EA ((a), (c), and (e)) or Tumor-No EA ((b), (d), (f)). Nikon ACT-1 (Nikon, Melville, NY) software was used to acquire images for processing, and tumor quantity and area were measured per section and totaled per animal using MetaMorph software (Molecular Devices, Sunnyvale, CA). EA-2X/3 significantly reduced the average tumor area present in the transverse lung sections ((a)/(c)/(e) compared to (b)/(d)/(f)). Scale bars represent ((a)-(b)) 500 *μ*m, ((c)-(d)) 200 *μ*m, and ((e)-(f)) 100 *μ*m.

As with many metastatic bone tumors, the potential for metastasis to other vital organs is extremely prevalent. In the present study, we focused our attention on the effect of EA-2X/3 treatment on metastasis from the hindpaw to the lungs, since this treatment regimen was the most effective in reducing tumor growth and reducing lymphatics within the tumor. Upon gross pathological examination, no visible lung metastases were observed in either the EA-2X/3 treated tumor mice or the untreated tumor mice prior to histological analysis at either 40 or 60 days after tumor implantation. To investigate whether EA treatment had an effect on mitigating osteosarcoma metastasis, we performed histological quantitative analysis to measure average lung tumor area and number of metastases at PID 40 and PID 60 in male mice treated with EA-2X/3 (see Figure 5).

**Figure 6:**
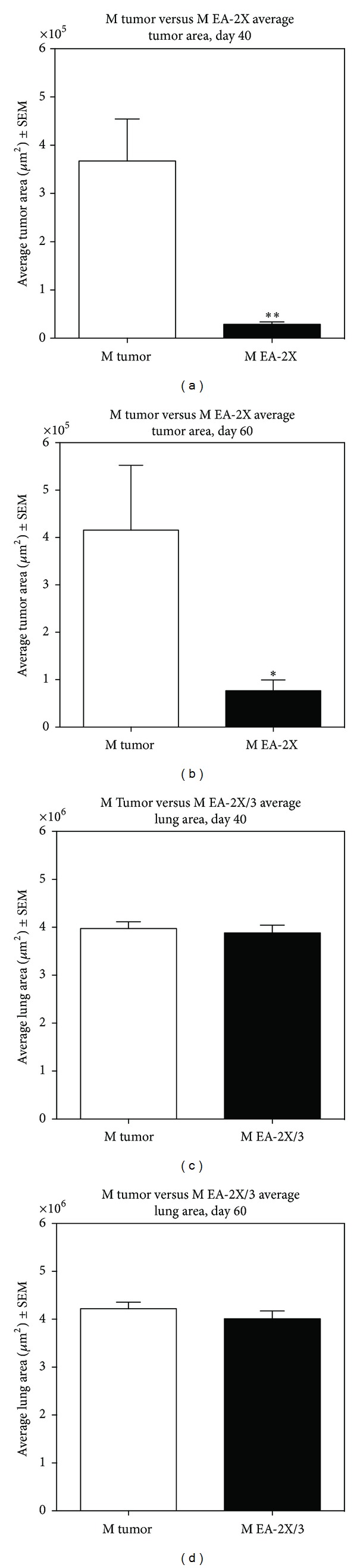
Graphs illustrating the effects of electroacupuncture (EA) given twice weekly beginning PID 3 (EA-2X/3) on the average metastatic tumor area within the lung ((a) and (b); *n* = 3–6/group) and on the average lung area ((c) and (d); *n* = 3/group) at PID 40 (a) and PID 60 (b). The average metastatic tumor area was significantly reduced in the EA-2X/3 group on both PID 40 and PID 60. EA-2X/3 had no effect on the average lung area. Sections were stained by H&E, data shown as mean ± SEM; *P < 0.05 or **P < 0.01 as compared to Tumor-No EA control.

A significant reduction in average lung tumor area was observed as early as PID 40 and was sustained to PID 60 when compared to the Tumor-No EA control group (Figures 6(a) and 6(b); *P < 0.05). Image analysis measurements for average tumor area on PID 40 were 367495 ± 86796 versus 28922 ± 4908; on PID 60 they were 415774 ± 136735 versus 76602 ± 22821, M tumor versus M EA/2X with EA-2X/3, respectively. This suggests that EA-2X/3 treatment reduces tumor metastases during both the early and later stages of tumor development.

When comparing the number of metastases across groups, EA-2X/3 treated animals did not show any significant differences in numbers of metastases compared to other groups. Thus, the total number of tumor metastases remained comparable between both EA-2X/3 and Tumor-No EA at both the PID 40 and PID 60 time points (data not shown) despite the fact that metastatic tumor area was significantly less in the EA-2X/3 group. To assure that the total lung area occupied by both lungs did not differ between the mice in the two groups, the average lung area from the tumor mice treated with EA-2X/3 was compared with the average lung area from the Tumor-No EA mice. No significant differences in average lung area (*μ*m^2^) were observed in either the EA-2X/3 or Tumor-No EA control group on PID 40 or PID 60 (Figures 6(c) and 6(d); *P* > 0.05). 

## 4. Discussion

 Using an osteosarcoma bone cancer model in the present study, we established the following novel findings: (1) EA can affect tumor growth and metastasis; (2) different EA regimens have differential effects on tumor growth; and (3) sex differences are evident in these effects. This has significant implications for the use of acupuncture to treat cancer patients for the relief of cancer pain or for the relief of the side effects of radiation or chemotherapy. Importantly this raises the issue of whether acupuncture can be used to slow tumor growth? Over the past decade, there has been a dramatic increase in the use of complementary and alternative medicines by cancer patients despite limited research available to support their use [6]. In this regard, acupuncture has been used to treat the side effects of cancer therapy as well as the fatigue, pain, sleep disturbances, and lymphedema associated with many types of tumors [1–3, 32, 33]. While there are a number of studies that have provided evidence for the antinociceptive, antifatigue, and antiemetic effects of EA in cancer patients [4, 33], there is only one case report that suggests that acupuncture may actually affect the tumor itself (by causing regression of human ductal carcinoma [34]). Moreover, there are no controlled studies in the literature that we are aware of that have evaluated acupuncture's effect on tumor metastasis and no studies that have examined potential sex differences in EA's effect on tumor growth. As alluded to above if acupuncture can in fact alter tumor growth and/or metastasis, then this would have a major impact on the use of acupuncture for treating cancer itself and could potentially require monitoring of its use for treating cancer associated symptoms like pain, nausea, and fatigue to assure that such treatment does not promote tumor growth or spread.

 With respect to the time course of treatment on tumor growth, none of the six EA regimens used in this study had any effect on tumor growth within the first 10 days following tumor cell implantation, despite that fact that in previous work we have demonstrated that these same regimens can alter tumor-induced pain as early as PID 3 [13]. This would imply that EA's effect on tumor pain is not associated with its effect on tumor growth during the first two weeks after tumor cell implantation. With respect to longer-term effects (after PID 10), we show that only EA given twice weekly beginning at either PID 3 or PID 5 significantly reduces tumor growth. Conversely, EA given either once weekly or twice-weekly starting at a later time point (PID 7) significantly accelerated tumor growth over the course of the study. This has several implications: (1) acupuncture treatment should be performed early in the course of tumor development in order to effectively reduce tumor growth; and (2) if acupuncture is begun later during tumor development, the possibility that it can enhance tumor growth and metastasis must be considered. While there are a number of studies that have examined the use of Chinese herbs and botanicals to treat cancer [35, 36], there are very few studies in the literature that have examined the effects of acupuncture on tumor growth or metastasis. Recently, Dehen [34] described a human case of ductal carcinoma in situ that regressed after treatment with acupuncture and Chinese herbs without surgery or chemotherapy. While this is only an “*n*” of one, it represents one of the first reports showing an effect of acupuncture and Chinese herbs on tumor growth. Using three different rodent cancer models, Lai and coworkers [37] applied EA concurrently to 3 separate acupoints including ST36 once per day for 15 days and demonstrated that this treatment significantly reduced gross tumor volumes in liver cancer, gastric cancer, and in a hypodermic tumor. While this study stimulated 3 acupoints daily for 15 days, the results are consistent with our finding that stimulating ST-36 twice a week starting at either PID 3 or PID 5 reduced tumor growth. Using a breast cancer model in mice, Liu and coworkers [38] similarly demonstrated that acupuncture treatment decreased mammary tumor volume. Finally, there is one report showing that acupuncture given at ST-36 can inhibit cancer cell division, but tumor growth was not measured as part of this study [8]. Collectively, these reports document that acupuncture can inhibit tumor growth and are consistent with our results obtained following application of EA twice per week beginning at PID 3 or 5. However, we were unable to find any studies in the literature documenting that acupuncture increases tumor growth, which was a surprising finding following stimulation of ST-36 once or twice per week beginning at PID 7.

Women are almost twice as likely to seek complementary and alternative health care compared to men, and they make up 60% and 70% of acupuncture claims [39, 40]. Thus, it is crucial to assess potential sex differences in acupuncture treatment, particularly since few studies have attempted to analyze such differences [41]. In this regard, it is worth noting that during acupuncture treatment, fMRI images of the brain indicate significant sex differences, suggesting that the efficacy of treatment might be different between men and women [42]. Moreover, following EA treatment, women demonstrate a significant increase in pain threshold, which is the opposite of what is found in men [43]. These differences reinforce the idea that the sex of the patient should be included as an important factor when assessing treatment outcomes. With respect to acupuncture, we have previously demonstrated that there are sex differences in acupuncture's effect on tumor pain [13]. Here we establish for the first time that there are also sex differences in EA treatment effects on bone cancer. While osteosarcoma tumor growth occurs in both male and female mice, both EA-1X/7 and EA-PRO administered to the ST-36 acupoint produced an increase in tumor growth rate in male, but not female mice. Moreover, EA-2X/7 treatment produced an increase in both male and female tumor growth, but this increase occurred earlier in female mice (PID 14). Conversely following EA-2X/3 treatment, decreases in tumor growth occurred earlier in male animals. Collectively, our data suggest that sex differences do exist with respect to EA treatment, and thus sex should be a factor in determining appropriate acupuncture regimens. 

In more recent years, evidence has accumulated indicating that there is a strong link between early inflammation and cancer [44–46]. This inflammatory cascade may be mediated in part by cytokines, chemokines, and other extracellular proteins that are involved in the malignant conversion of these cells. These components can promote metastatic tumor phenotypes including angiogenesis, accelerated growth, and malignancy. Our previous work indicated that acupuncture has an anti-inflammatory effect on bone cancer and is able to reduce neutrophil influx to the tumor site [13]; the current work indicates that acupuncture can also modify blood vessel, lymphatic, and nerve innervation density within the core and/or periphery of the bone tumor. While the exact acupuncture associated mechanism leading to a reduction in lung metastasis remains to be defined, it is likely that the EA-induced reduction in blood vessels at the tumor core and the reduction in lymphatics in the tumor periphery contribute to the reduced lung metastases. While previous studies have shown that acupuncture can affect angiogenesis [24], blood flow [47], and microvascular ultrastructure [48], this is the first study to show an acupuncture-induced decrease in blood vessels at a bone tumor site as well as a decrease in lymphatics in the periphery of the tumor. Ouyang and colleagues [49] demonstrated that electroacupuncture has the ability to reduce vascular endothelial growth factor in the peripheral blood of patients with rheumatoid arthritis, and this may be one mechanism by which EA is able to reduce blood vessel density in bone tumors. We also found that EA significantly decreased the density of nerve fibers innervating the tumor, but the potential relationship between this and tumor growth or metastasis remains to be determined.

## Figures and Tables

**Table 1:** Summary of the total number of male and female animals used for each experiment.

Assay	Treatment type	Male (*n*)	Female (*n*)
Electroacupuncture			
	EA-1X/7	10	8
	EA-2X/3	12	9
	EA-2X/5	7	7
	EA-2X/7	7	7
	EA-Pro	6	8
	EA-Once/1	11	11
	Tumor-No EA	10	10
	Saline	7	7
Immunohistochemistry			
	LYVE-1	3	0
	CD31	3	0
	PGP9.5	3	0
Lung histology			
	EA-2X/3	10	0
	Tumor-No EA	11	0

Total:		100	67
